# Mesocarnivore community structure under predator control: Unintended patterns in a conservation context

**DOI:** 10.1371/journal.pone.0210661

**Published:** 2019-01-17

**Authors:** Gonçalo Curveira-Santos, Nuno M. Pedroso, Ana Luísa Barros, Margarida Santos-Reis

**Affiliations:** 1 cE3c - Centre for Ecology, Evolution and Environmental Changes, Faculdade de Ciências, Universidade de Lisboa, Campo Grande, Lisboa, Portugal; 2 CENA, Universidade de São Paulo, Piracicaba, São Paulo, Brazil; Charles Darwin University, AUSTRALIA

## Abstract

Across the Mediterranean, conservation programmes often operate concomitantly with hunting interests within game-lands. Carnivore guilds lie at the interface between contrasting management goals, being simultaneously fundamental components of ecosystems and targets of predator control to reduce predation on game species. Here, we evaluate the composition and spatial structure of a mesocarnivore community in a protected area of Southeast Portugal, with high economic investment in conservation and significant hunting activity. Between June and August 2015, we deployed 77 camera-traps across a ~80 km^2^ area. We report on interspecific disparities in mesocarnivore occupancy and associated environmental determinants. Contrasting occupancy states suggest an unbalanced community, biased towards the widespread occurrence of the red fox *Vulpes vulpes* ($\hat{\psi }=0.92\pm 0.04$) compared to other species (stone marten *Martes foina*, European badger *Meles meles*, Egyptian mongoose *Herpestes ichneumon*, common genet *Genetta genetta*, and Eurasian otter *Lutra lutra*) exhibiting spatially-restricted occupancy patterns ($\hat{\psi }/naïve\;occupancy<0.35$). The feral cat *Felis silvestris catus* was the exception ($\hat{\psi }=0.48\pm 0.11$) and, together with the stone marten, exhibited a positive association with human settlements. These findings are consistent with theoretical predictions on how mesocarnivore communities are shaped by the effects of non-selective predator control, paradoxically favouring species with higher population growth rates and dispersal abilities, such as the red fox. Our results reinforce the need to understand the role of predator control as a community structuring agent with potential unintended effects, while exposing issues hindering such attempts, namely non-selective illegal killing or biased/concealed information on legal control measures.

## Introduction

Unlike in many parts of the globe, where protected areas aim to preserve ‘pristine nature’ free of human interference, Mediterranean Europe is dominated by inextricably linked human-shaped and natural systems [1]. In many protected areas, conservation programmes and nature protection legislation act concomitantly with multiple human activities and exploitation of natural resources. This is the case of the Iberian Peninsula, where protected areas often spatially overlap with game-lands. Hunting estates or game reserves cover a sizeable proportion of non-urbanized land and hunting for sport or leisure is a key cultural activity with high socio-economic relevance in the region [2]. Both conservationists and hunters share common goals in the preservation of valuable habitats [3] and restoration of small-game populations, namely the European rabbit *Oryctolagus cuniculus* and red-legged partridge *Alectoris rufa*, that are also important food resources for predators [4,5]. Rabbits, in particular, are keystone prey species for several predators in Mediterranean ecosystems [6] and a fundamental element of the Iberian game-based economy [7]. Recovery efforts for this species have been greatly associated with management interventions within game estates [6,8]. Nevertheless, there is rarely agreement on how local natural values should be managed. For instance, conflict arises when culling, as a method for controlling predator species, is undertaken to protect hunting interests, having potential detrimental effects on target predator populations and other vulnerable species [5,9–12].

Carnivore guilds (i.e. order *Carnivora*), and particularly mesocarnivore communities, lie at the interface between these two contrasting management practices. From a conservation standpoint, carnivore species are considered fundamental components of natural ecosystems [13,14], providing several relevant ecological services, such as top-down regulation of lower trophic levels, seed dispersion or disease mitigation [15–19]. Hence, maintenance of a healthy carnivore guild is of great conservation relevance [13,20]. From the perspective of hunting interests, the major focus for carnivore population management via predator control methods [21] is to reduce game predation, thereby enhancing the harvestable fraction of predator-sensitive species [5,22–24]. Therefore, predator control is deemed by game managers as an essential tool for increasing their economic income from hunting activities [7,21]. According to Delibes-mateos et al. [12], the perception of predators being too abundant is most often based on predator observations or direct observation of predation events, instead of proper quantification. In Iberia, red fox *Vulpes vulpes* and Egyptian mongoose *Herpestes ichneumon* are considered hunting species and both may be legally targeted for predator control actions (Law Decree n.° 202/2004).

Although extremely relevant, the impacts of game management and predator control efforts on Iberian mesocarnivore community structure and composition are poorly understood (but see [4,5]) and empirical data on community shaping effects of predator control measures is still scarce (see [25,26]). Impacts of culling activities on predators and the effectiveness of removal generally vary according to the selectivity, intensity and duration of the methods applied, as well as on the target species biology, such as life-history traits, and behaviour or habitat preferences [27]. Unless predator control is enduring, exhaustive and extensive in scope, the results are often very limited, frequently failing to induce long-term population declines of target species [28–31] while negatively affecting more sensitive predators, due to the low selectivity of control methods applied [12,27,32]. Model simulations of Mediterranean mesocarnivore populations exposed to non-selective predator control in the presence of intraguild competition suggest species with a low intrinsic growth rate can experience decreased population size or become locally extinct, whereas species with a higher reproductive rate, can maintain or even exhibit population increases [25,26]. Such effect is mainly attributable to vacant competitive niche space, a fast recovery rate and high prey availability. Simultaneously, the high prey densities promoted in hunting estates through supplementary feeding (e.g. pastures, feeding stations) and restocking might also affect local mesocarnivore community structure. Increased prey abundance provides improved foraging conditions for predators that consume small game species (e.g. [5]). Those same conditions might attract predators that are later culled, thus creating population sinks with consequences for biodiversity conservation and community dynamics. Such patterns raise concerns for harmful and unintended consequences at the ecosystem level and fall under a growing body of evidence on context-dependent ecological roles of predators in anthropogenic landscapes [33,34]. An understanding of how anthropogenic practices shape carnivore assemblages is of great conservation interest and a critical issue for effective game management within protected areas.

Here, we evaluated the composition and spatial structure of a mesocarnivore community in Guadiana Valley Natural Park (GVNP), Southeast Portugal. This protected area was created in 1995 and features high economic investment in conservation (European-funded LIFE projects, endangered species management plans, habitat and species restoration programmes) and significant hunting activity (about 86% of the land is included in hunting estates). The GVNP is subjected to legal predator control, through box-traps and predator-targeted hunts, but there are also evidences of widespread illegal non-selective culling (e.g. snaring, unauthorized box-traps; pers. obs.). Specific objectives for this study were two-fold. Firstly, we aimed to evaluate inter-specific variation in occupancy, as a surrogate for abundance, among local mesocarnivores, considering two hypotheses: *i*) mesocarnivores species exhibit spatially restricted occupancy patterns due to ongoing, non-selective, predator control [4,5]; *ii*) the red fox, despite being the main target of predator control, is the least affected species exhibiting marked differences in occupancy in comparison to sympatric mesocarnivores [25–27]. Secondly, we intended to ascertain the effect of environmental factors underlying the occupancy estimates obtained, hypothesising that species-specific occupancy patterns are influenced in a non-exclusive manner by: *iii*) landscape structure, reflecting previously described species-habitat relationships [35,36]; *iv*) prey availability, following a positive effect of subsidized small game populations [5,6]; and *v*) anthropogenic disturbance, denoting avoidance behaviours relative to human infrastructures and hunting management [5,37]. We discuss our findings in the context of previous theoretical and empirical-based predictions on the community shaping effect of predator control in Mediterranean environments and elaborate on factors hindering quantitative evaluations of predator control role in altering carnivore communities.

## Study area

**Fig 1:**
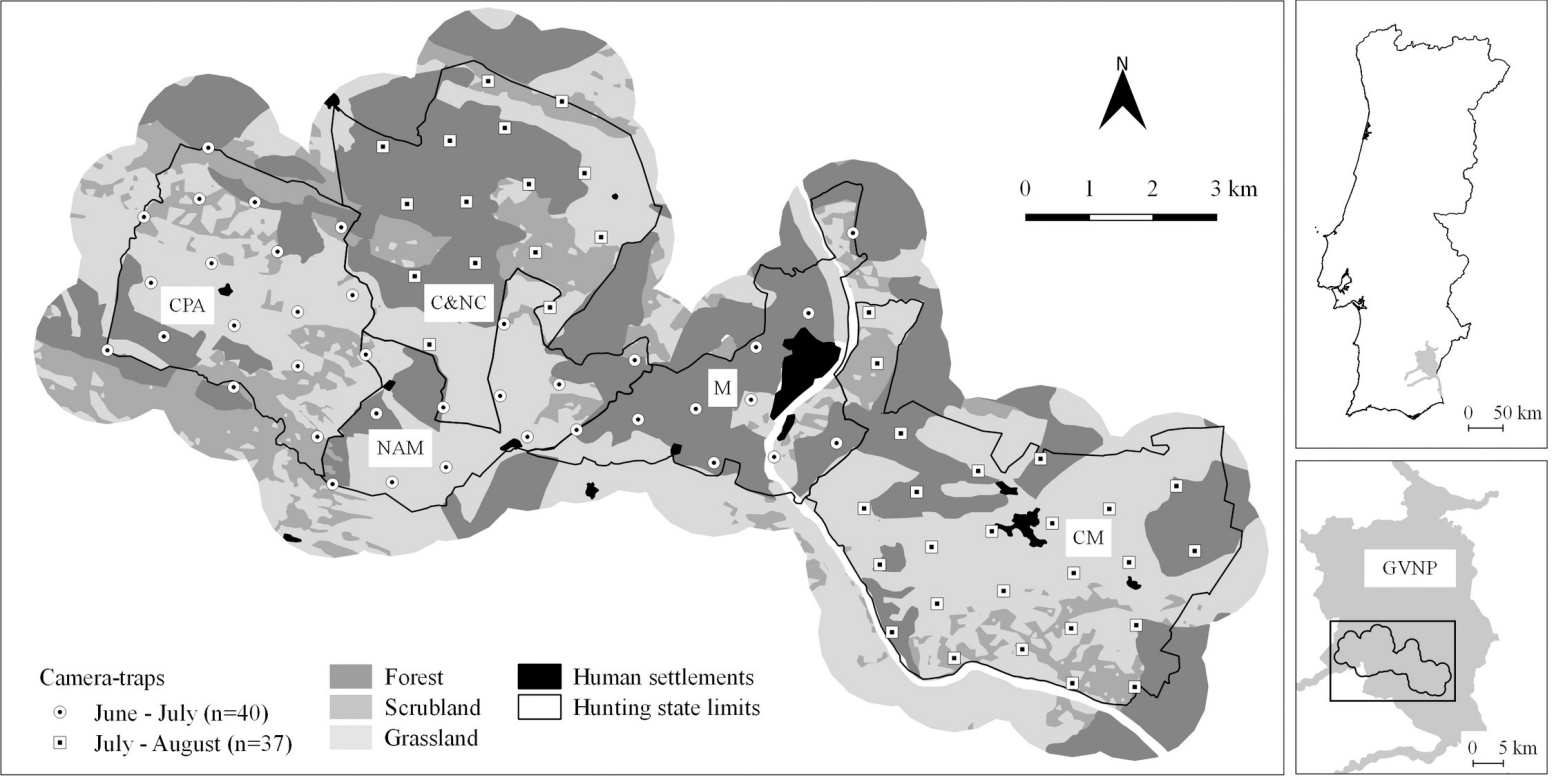
Study area location, hunting estates surveyed (CPA, NAM, C&NC, M and CM), land-cover types and camera-trap stations in the Guadiana Valley Natural Park.

This study was conducted in a ~80 km^2^ area within GVNP (total area: ~700 km^2^; 37°42’N 07°39’W) in Southeast Portugal (Fig 1). Located in the Guadiana river basin, GVNP constitutes an important ecological corridor in Southern Portugal, harbouring several endangered species in Europe, such as the Iberian Lynx *Lynx pardinus* and the Spanish Imperial Eagle *Aquila adalberti* ([38]; Life Imperial LIFE13 NAT/PT/001300). The study area comprised five hunting estates with different intensities of small game population reinforcement (European rabbit and red-legged partridge) and predator control. The landscape is highly heterogeneous with cereal croplands and pastures interspersed with scrubland patches and forested systems dominated by *Pinus pinea/pinaster* and holm oak *Quercus ilex* (Fig 1). Permission to conduct field work in each hunting estate was given by the hunting estates’ managers to the LIFE+ Nature project “Conservation of the Spanish Imperial Eagle (*Aquila adalberti*) in Portugal” (LIFE13 NAT/PT/001300).

## Methods

### Carnivore data

Camera-trapping was used to evaluate the community composition and occupancy patterns of local mesocarnivores [39]. Between June and August 2015, 77 camera-trap stations (Moultrie M-990i Gen 2 Game Camera) were regularly spaced across the study area, each operating for 24 consecutive days in a two-phased rotation scheme (Fig 1). The summer period was selected as higher carnivore densities and juvenile activity can promote increased detection probabilities [35]. Trapping stations were positioned using a 1x1 km regular grid guideline (average distance = 900 m; SD = 87 m; max = 1275 m; min = 766 m), so that the entire study area was surveyed. Cameras were placed along dirt roads to maximize target species detectability [40], attached to trees or to wooden stakes at ~30 cm above ground [41]. Each camera was programmed to operate for 24 h per day and to take three sequential photographs once triggered (at 1 s intervals), to facilitate species identification, with minimum delay between triggers (5s). Stations were visited halfway through the survey to check cameras and to replace batteries/memory cards. Camera-trapping is a non-invasive method and, therefore, no ethical permits were required to carry out this project.

Each photograph was identified to the species level. Because European wildcats *Felis silvestris silvestris* and feral or free-roaming domestic cats *Felis silvestris catus* (hereafter feral cat) co-occur in our study area, the species in each “cat” record was identified based on the most diagnostic phenotypic traits; namely, the “7-score pelage characters” [42]. We adopted a conservative approach and identified as domestic all cats without a clear wild morphotype as these could be potential hybrids [43].

### Occupancy modelling

Carnivore species occupancy (*ψ*; probability of the species occupying a site) was estimated under a maximum likelihood-based approach, explicitly accounting for imperfect detection (*p*; probability of the species being detected if present) [44]. Species-specific binary detection histories (0 for non-detection and 1 for detection) were constructed for each trapping-station and collapsed into 6-day sampling occasions; maximizing both the number of sampling occasions and species detectability rates. Occupancy matrix and covariate data are deposited in Dryad (doi:10.5061/dryad.36g41fd). With 24-day surveys we assumed populations closed to occupancy changes, thus allowing the use of single-season occupancy models to estimate species-specific occupancy states and to assess the influence of individual environmental covariates. The single-season occupancy model [44,45] collects information on temporally-repeated sampling occasions over several sites (i.e. camera-trap stations) to construct a likelihood estimate based on probabilistic arguments that correct false-negative surveys by estimating a probability of detection. Estimates of site occupancy thereby provide a useful framework for exploring distribution patterns and habitat associations of elusive species such as most carnivores.

For each carnivore species, a suite of candidate models for both detection (p) and occupancy (ψ) parameters was compared following a two-stage approach for modelling procedures. In the first stage, detection probability was modelled as a function of environmental covariates while keeping occupancy constant (i.e. p(‘covariate’), ψ(.); e.g. [46]). The best-fitting detectability model was then combined with candidate models representing plausible biological hypotheses explaining carnivore species’ occupancy probabilities.

Potential covariates affecting detection probability, proxy for activity, were chosen following the work of Monterroso et al. [47], on the same species and within the same region, and complemented with the inclusion of disturbance covariates associated with anthropogenic infrastructures. Human settlements and paved roads, widespread throughout our study area, can induce avoidance behaviours [37,48], which may affect not only the presence of a species but also the activity of individual animals in their proximity. In total, six site-specific covariates were tested in univariate candidate models of detection probability: (1) habitat type; (2) elevation; (3) slope; (4) distance to water sources; (5) distance to nearest paved road; and (6) distance to nearest human settlement (Table 1). Additionally, a null model without covariates and constant ψ and p (i.e. p(.), ψ(.)) was included in the candidate model set for detectability.

**Table 1:** Covariates used to assess target carnivore species occupancy patterns in the five studied hunting estates in the Guadiana Valley Natural Park; *Ψ* —occupancy probability, *p*—detection probability.

Covariate	Type	Code	Description	Units	Data range (min-max)
**LANDSCAPE**					
Micro-scale habitat	*p*	Hab	Habitat type assigned to the precise location of each camera-trap station, classified into three major structural types (forest, shrub, and grassland) from vegetation GIS coverage, with a spatial resolution of 30 m.	factorial	1–3
Forested habitat	*Ψ*	Forest	Forested systems of stone pine *Pinus pinea* L. and holm oak *Quercus ilex* L.		
Mediterranean scrublands	*Ψ*	MScrb	Areas dominated by tall shrubs (>1m) of *C*. *ladanifer* and *C*. *monspeliensis*.	% cover	0–88
Grasslands	*Ψ*	Grass	Cereal cultures, fallows or pastures without shrub or tree cover.	% cover	0–1
Landcover diversity	*Ψ*	Ldiv	Simpson’s landscape diversity index.	0–1	0–0.76
Distance to water sources	*Ψ*, *p*	DistWS	Linear distance between camera-trap station and nearest water source (i.e., watercourse or reservoir).	meters	13–898
Slope	*Ψ*, *p*	Slp	Slope in degrees from DEM with a spatial resolution of 30m; assigned locally to each station (*p*) or averaged within a 500m buffer (*Ψ*).	degrees	0.93–25.65 (30m); 4.10–14.04 (500m)
Elevation	*Ψ*, *p*	Ele	Elevation above sea level with a spatial resolution of 30m; assigned locally to each station (*p*) or averaged within a 500m buffer (*Ψ*).	meters	24–262 (30m); 38–207 (500m)
**PREY**					
Rabbit encounter rate	*Ψ*	Rabbit	Number of independent rabbit records in each camera-trapping station per 100 trapping days.	#	0–353.57
Red-legged partridge encounter rate	*Ψ*	Pat	Number of independent red partridge record in each camera-trapping station per 100 trapping days.	#	0–157.14
**DISTURBANCE**					
Distance to nearest paved road	*Ψ*, *p*	DistR	Linear distance between camera-trap station and nearest road.	meters	24–2138
Distance to nearest human settlement	*Ψ*, *p*	DistSet	Linear distance between camera-trap station and nearest human settlement (i.e. group of inhabited buildings).	meters	106–2369
Hunting estate	*Ψ*	HEstate	Blocking factor with 5 levels (one per hunting estate) to characterize overall differences in management strategies.	factorial	1–5

For ψ, tested environmental covariates were grouped into three categories: (1) Landscape; (2) Prey; and (3) Disturbance (Table 1). Landscape covariates were determined based on cover of local main habitats, reclassified into three major structural types—Forest, Scrubland, Grassland—and expressed as the proportion of cover within a circular buffer of 500 m radius around each station. Furthermore, an index of landcover diversity was computed according to the richness and relative proportions of different patches within the buffer. Landscape metrics were obtained from vegetation geographic information system coverage available for GVNP, with a spatial resolution of 30 m (LIFE08 NAT/P/000227) and calculated in Quantum GIS (version 2.10.1 Pisa) [49]. Data on elevation and slope was extracted from the Advanced Spaceborne Thermal Emission and Reflection (ASTER) radiometer global digital elevation model (GDEM, http://www.gdem.aster.ersdac.or.jp). Distance to water sources was obtained by measuring the linear distance from the camera-trap station to the nearest watercourse or reservoir. Camera-trapping capture rates of small game species, i.e. European rabbit and red-legged partridge, were used to quantify relative game-prey availability and interpreted as an index of prey encounter rate, expressed as number of independent camera records (> 1 h interval) per 100 trap-days [50]. Prey measures were not interpreted as abundance indexes but rather as likelihoods of carnivore-prey encounters at each camera-trap station. Disturbance variables were characterized by linear distances to the nearest roads and human settlements. Positions of roads and human settlements were obtained from a 1:10,000 digital map. Each station was assigned to the corresponding hunting estate, generating a blocking factor with five levels (one per hunting estate) to account for differences in management strategies not reflected in above described covariates, specifically on the intensity of predator control methods applied.

To avoid the risk of model over-parameterization that could reduce the precision of each species occupancy estimates, we did not consider candidate models comprising combinations of several covariates [51] and assessed only univariate candidate models, i.e. one covariate per *p* and ψ model (e.g. p(habitat), ψ(Scrubland)). The exceptions were models composed of two covariates that characterized overall prey abundance (ψ(Rabbit+Pat)) and disturbance (ψ(DistR+DistSet)). Prior to the analysis, pairwise covariate relationships were assessed using Spearman's correlation coefficients, to ensure that no highly correlated variables (r ≥ 0.7) were incorporated in the models and avoid multi-collinearity [52]. Continuous variables were standardized to z scores to facilitate coefficient interpretation and comparison [53].

The Akaike Information Criterion corrected for small sample sizes (AICc) was used to rank candidate models [54]. Only models with ΔAICc values ≤ 2, comparatively to the most parsimonious model in the set, were considered to estimate species-specific occupancy states ($\hat{\psi }\pm SE$) and to identify important environmental covariates. In cases where several top-ranked models were identified, a model averaging approach was adopted to draw inferences and to compute site-occupancy estimates [54]. The effects of site-specific covariates on p and ψ were evaluated via a logit-link function and their effects were considered well-supported when 90% unconditional CI’s of averaged *β* estimates did not overlap zero [44]. Goodness-of-fit of the most parsimonious models was tested using a Pearson chi-square statistic and parametric bootstrapping (1000 samples) [55]. Occupancy models were implemented using the ‘unmarked’ package [56] in R statistical software V. 2.15.1 [57]. Lastly, carnivore-specific occupancy maps were generated based on averaged site-occupancy estimates by inverse distance weighted interpolation (i.e. IDW; [58]), producing spatial interpolation surfaces of carnivore occupancy probability for the entire study area.

## Results

Our camera-trapping effort generated a total of 1925 effective camera-trap days and a total of 902 independent carnivore captures (i.e. 47.3 carnivore captures/100 trap-days) of seven carnivore species (Table 2): red fox, feral cat, stone marten *Martes foina*, European badger *Meles meles*, Egyptian mongoose, Common genet *Genetta genetta*, Eurasian otter *Lutra lutra*. The red fox alone accounted for ~80% of carnivore captures.

**Table 2:** Community composition, trapping success (species ranked per decreasing order), and number of occupied sites (i.e. sites with at least one detection) by carnivore species in the five studied hunting estates in the Guadiana Valley Natural Park, as obtained from camera-trapping campaigns between June and August 2015.

Species	Captures	Captures / 100 trap-days	# occupied stations
Red fox *Vulpes vulpes*	722	37.9	71
Feral cat *Felis silvestris catus*	113	5.9	29
Stone marten *Martes foina*	28	1.5	17
Egyptian mongoose *Herpestes ichneumon*	18	0.9	8
European badger *Meles meles*	13	0.7	10
Common genet *Genetta genetta*	4	0.2	3
Eurasian otter *Lutra lutra*	4	0.2	2
**Total**	902	47.3	74

**Fig 2:**
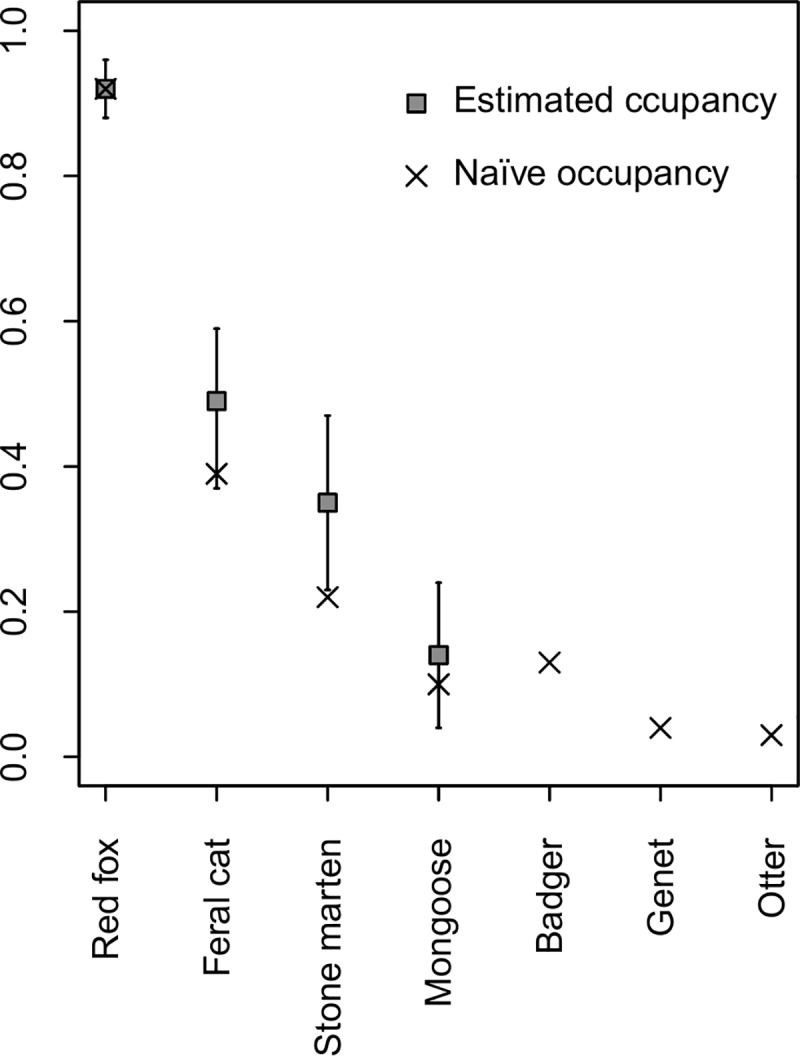
Carnivore species-specific naïve and estimated occupancy states in the five studied hunting estates of the Guadiana Valley Natural Park, as calculated from camera-trapping campaigns between June and August 2015.

**Fig 3:**
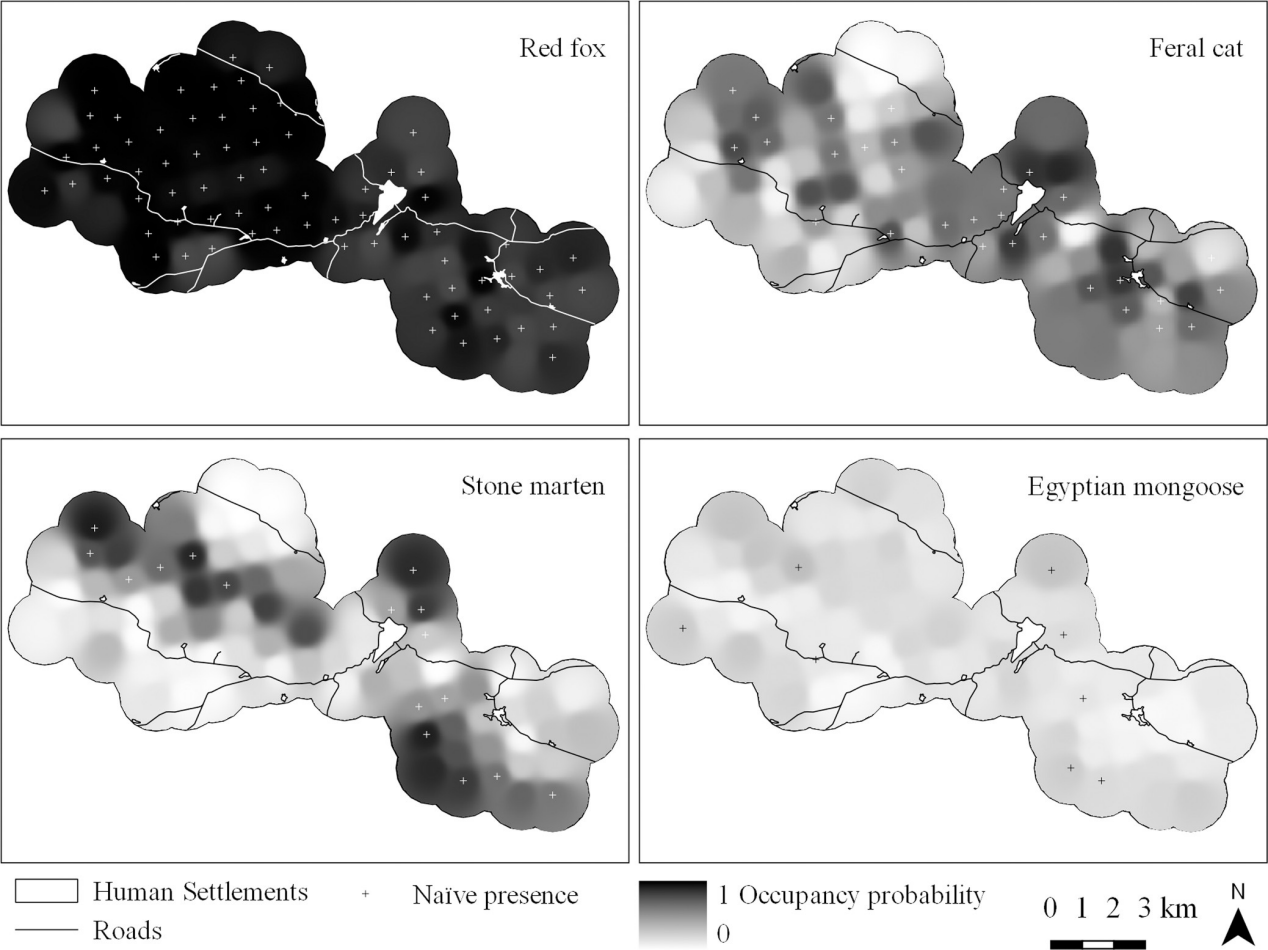
Occupancy probability surfaces of carnivore species in the five studied hunting estates in the Guadiana Valley Natural Park.

Occupancy models, explicitly accounting for imperfect detection, exhibited contrasting occupancy states among the community members considered for analysis (Average $\hat{\psi }=0.48$; SD = 0.29; Max = 0.92 [red fox]; Min = 0.14 [Egyptian mongoose]) (Figs 2 and 3). For the European badger, the common genet and the Eurasian otter, we considered only naïve occupancy states, that is, the fraction of camera-trapping stations where the species was actually detected, since, when detection probability falls below 0.15, occupancy models can generate unreliable parameter estimates and fail to distinguish sites where the species is truly absent or merely poorly detected [45].

Modelled occupancy patterns were mainly dependent on environmental predictors related to human disturbance–with well supported effects for three species (feral cat, stone marten and Egyptian mongoose)—while prey availability and landscape structure covariates influenced one species each, respectively the red fox and the Egyptian mongoose (Table 3). Carnivore detectability was generally lower in steeper areas or influenced by the distance to the nearest water source. Goodness-of-fit tests on top-ranked models of all species indicated that these fitted the data adequately (p-values ≥ 0.096).

**Table 3:** Model selection results (ΔAICc ≤ 2) for carnivore species occupancy in the five studied hunting estates in the Guadiana Valley Natural Park, as estimated from camera-trapping data; Ψ —occupancy probability, p—detection probability; Covariate abbreviations are presented in Table 1.

Species	Model	K	AICc	ΔAICc	AICcw	GOF p-value^a^
Red fox	Ψ(Rabbit) p(Slope)	4	339.46	0.00	0.48	0.096
Feral cat	Ψ(DistSet + DistR) p(Slope)	5	243.36	0.00	0.88	0.577
Stone marten	Ψ(DistSet + DistR) p(DistWP)	5	146.16	0.00	0.74	0.382
Egyptian mongoose	Ψ(Ldiv) p(DistWP)	4	87.47	0.00	0.26	0.425
	Ψ(Grass) p(DistWP)	4	88.86	1.40	0.13	0.504
	Ψ(Slope) p(DistWP)	4	88.96	1.49	0.13	0.449
	Ψ(DistR) p(DistWP)	4	88.97	1.50	0.12	0.459

^a^ Goodness-of-fit test using the Pearson chi-square statistic; p-values ≤ 0.05 indicate poor model fit [55].

The red fox was the species with highest capture rate (24 times the average capture rate of the remaining community members; Table 2) and occupied nearly all survey sites across the study area. It was detected at 71 sites, corresponding to a naïve site occupancy of 92% (Figs 2 and 3). Detectability of the species was associated with local scale slope, decreasing in steeper areas (β_1_ = -0.23±0.13), and the probability of site occupancy was positively correlated with relative local rabbit abundance (Tables 3 and 4). Occupancy estimates ($\hat{\psi }=0.92\pm 0.04$) did not differ from naïve occupancy (Fig 2).

**Table 4:** Beta coefficient estimates on the logit scale and standard error (SE) for covariates contained in the best models of carnivore occupancy in the five studied hunting estates in the Guadiana Valley Natural Park, as estimated from camera-trapping data.

Species	Slope	Grass	Ldiv	Rabbit	DistSet	DistR
Red fox	-	-	-	1.95 (1.12)^a^	-	-
Feral cat	-	-	-	-	-2.12 (0.77)^a^	1.79 (0.70)^a^
Stone marten	-	-	-	-	-1.84 (1.05)^a^	3.01 (1.28)^a^
Egyptian mongoose	0.80 (0.48)^a^	-0.94 (0.51)^a^	1.24 (0.63)^a^	-	-	0.83 (0.43)^a^

^a^ Indicates a well-supported effect (i.e. estimated 90% CIs for unconditional β coefficients do not overlap zero).

Feral cat detection probability was negatively influenced by increasing slope near the camera-station (β_1_ = -0.86±0.25). The accommodation of detection variation in the occupancy modelling contributed to a 10% increase in the estimated proportion of occupied sites ($\hat{\psi }=0.48\pm 0.11$) compared to the naïve value of 38% (i.e. detections at 29 stations) (Figs 2 and 3). Spatial variation in the feral cat occupancy probability was determined by contrasting effects associated with distances to disturbance variables (Table 3); it was negatively related to distance to human settlements (DistSet), but positively related to the distance to the nearest road (DistR), both well-supported effects (Table 4).

Modelling of stone marten detectability originally reported distance to the nearest road (β1 = 1.00±0.25) as being the best predictor of variation in detection probability. However, when hierarchically coupled with the occupancy likelihood component, the model led to non-convergent and unreliable parameter estimates (very high standard errors). Hence, we removed this model from the candidate set and repeated the model selection procedures. The distance to nearest water sources was the main predictor of stone marten detectability (β_1_ = -0.53±0.39), although with only a marginally supported effect. Similar to feral cats, distance to human settlements (negative effect) and distance to nearest paved road (positive effect) described the key drivers of stone marten occupancy (Tables 3 and 4). The estimated proportion of occupied sites was 35% ($\hat{\psi }=0.35\pm 0.12$), representing a 13% increase relative to naïve states derived from a confirmed presence at 17 sites (Figs 2 and 3).

Egyptian mongoose was only captured at eight sites, representing the species with the lowest naïve occupancy (0.10) among those selected for analysis (Figs 2 and 3). Distance to nearest water sources had a positive effect on this species’ detection probabilities (β1 = 1.32±0.51). The most parsimonious models for Egyptian mongoose occupancy described the influence of landscape and disturbance variables (Table 3). Egyptian mongoose occupancy was positively associated with steeper terrain (i.e. increased slope) and landscape diversity but was negatively related to the percentage of grassland (Grass) cover within the buffer area. Like other species, mongoose occupancy likelihood decreased with proximity to roads (Table 4). The final averaged occupancy estimate was $\hat{\psi }=0.14\;(\pm 0.10)$, translating into a 4% difference from the naïve value (Fig 2).

## Discussion

In this study, we assessed the community composition, spatial occupancy patterns and underlying drivers of a Mediterranean mesocarnivore guild exposed to predator control in a landscape of conservation interest. Variation in species-specific occupancy states across our study area revealed large interspecific disparities (hypothesis *i*, *ii*) and variable effects of local environmental factors, with species occupancy mostly conditioned by disturbance variables (hypothesis *v*) and less by landscape structure and prey abundance (hypothesis *iii*, *iv*).

The list of species recorded in this study—six wild and one domestic—represents ~60% of mammalian carnivores potentially occurring in the region [59] and resembles those reported by Monterroso et al. [47], who conducted camera-trap surveys in a similar sized area within GVNP, differing only in the non-detection of the European wildcat and the detection of the Eurasian otter. The non-detection of the wildcat may be a result of our conservative approach to distinguish it from its domestic counterpart based solely on morphological traits [42], although most of the photographed individuals exhibited striking domestic pelage and none displayed a clear wild morphotype. Unsurprisingly, we did not detect the Iberian lynx, an “Endangered” species [60] recently re-introduced into the region (LIFE10NAT/ES/570) but still absent from the study area at the time of our study [61].

Overall occupancy estimates indicate a near two-fold difference between the red fox and the second-most common species, i.e. the feral cat, with even greater difference when compared with the remaining community members that occupy only a restricted proportion of the landscape ($\hat{\psi }/naïve\;occupancy<0.35$, hypothesis *i*; see Fig 2). Although our relatively short survey period and camera placement strategy might underestimate the presence of species with fewer detections and for which occupancy modelling was not possible to implement [62], or fail to detect the presence of species with specific patterns of habitat use (such as those associated with riparian habitats, e.g. polecat, otter; [63]), our comprehensive coverage of the landscape supports their restricted occurrence. These differences likely reflect disparities in local abundance given the intrinsic abundance-occupancy relationship at low population densities [64,65]. Additionally, we recorded at least one individual fox per 1 km^2^ grid square sampled using molecular analysis of red fox scats, collected in 500 m foot transects simultaneously to the camera-trapping campaigns (44 individuals confirmed from 31 transects with molecular assigned red fox scats; unpublished data). This evidence further supports the claims of a high-abundant fox population.

The shared importance of human-driven disturbance variables on target species is noteworthy within the context of a protected area. Feral cat and stone marten occupancy was explained by positive associations with human settlements and avoidance of paved roads. The dependence of free-ranging domestic cat populations on human settlements in Mediterranean areas has been previously described, being a key determinant of presence, abundance and space use patterns [66]. This is of conservation concern, as feral cats living or spending time in natural areas have access to rare, endangered and/or economically-valuable prey, may interbreed with wildcats, and increase the transmission of zoonotic diseases [43,67–69]. This pattern contrasts with that found for wildcats, that feed mostly on wild rodents and rabbits (e.g. [70]) and are sensitive species to human disturbance [71]. The population of feral cats in our study area is not currently managed. Future strategies to reduce this species impacts should target human settlements to reduce its spatial spread and avoid access to human refuse [66]. Stone martens are generalist carnivores that can be found in open areas, deciduous woodlands and forests, forest edges and mosaic habitats [72,73], as well as occupying towns and villages [74,75]. The negative response to road proximity exhibited by both these species, and shared by the Egyptian mongoose, might be a result of avoidance behaviours induced by wildlife–vehicle-collisions, a major factor of carnivore mortality in Mediterranean environments [37]. Conversely, the reduced importance of landscape descriptors is surprising since, for spatially-restricted species, stronger responses to habitat features are expected. Only the mongoose displayed such associations, with reduced occupancy probabilities in open grasslands and a preference for steeper areas and more complex habitats. These patterns are consistent with previous findings for the species reporting increased use of habitat mosaics [36,76]. The mongoose’s positive selection for steeper areas, usually associated with understory cover, as opposed to open pastures, may be due to its preference for sheltered environments where persecution by humans is presumably lower. Nevertheless, caution should be exercised in interpreting these patterns given the extremely low occupancy of this species.

When compared with other studies reporting data on Iberian mesocarnivores’ occupancy [35,36,47,77–81] (see S1 Text), the contrasting occupancy states we observed indicate an unbalanced predator community, biased towards the occurrence of the generalist red fox. Red fox’s occupancy was amongst the highest recorded across comparable studies of Iberian mesocarnivores (average naïve occupancy = 0.68±0.26; S1 Table), while exhibiting the largest interspecific disparities with sympatric species (S2 Table). These patterns are consistent with theoretical and empirical predictions on the community shaping effect of non-selective and spatially-variable predator control in Mediterranean environments [4,25–27,82]. Although we could not establish causality within the scope of this study (as discussed further below), model simulations of population dynamics within hypothetical mesocarnivore communities subjected to non–selective control and intra-guild competition suggest that even under moderate control, the red fox can exhibit population increases due to its higher intrinsic growth and immigration rates [83], whereas sympatric species having lower *r* values are negatively affected [25,26]. These theoretical predictions are supported by the patterns observed in this and other studies in landscapes managed for predator control, with control programs often proving ineffective at significantly reducing red fox density (e.g. [30,84]). This is particularly relevant considering the red fox is the main consumer of small game among our target species (mainly rabbit; [84,85]); as supported by local rabbit abundance as the single driver of red fox occupancy in our models. In contrast, when non-selective control is applied, either due to the method used or human release/kill decisions after capture [12,32], more sensitive and protected species such as badgers and martens tend to be locally rare [4,27,82]. The long-term absence of larger native predators, such as the Iberian lynx [61], and the consequent release of foxes may contribute to the observed community structure [86]. Such patterns raise concerns for harmful and unintended consequences at the ecosystem level and for species of conservation concern or economic value, failing to comply with both conservation and hunting goals. The restricted local distribution of several mesocarnivore species may reduce predatory and competitive pressures upon unwanted species (e.g. pest rodents; [87]), while the paradoxical red fox’s widespread occurrence may increase the predation levels upon species of conservation (e.g. ground-nesting farmland birds; [78]) and hunting interest [88].

Since our research took place in a unique landscape of high conservation and economic relevance, this study highlights the need for a better understanding of the mechanisms shaping community composition and structure in game managed landscapes. Nevertheless, inferences beyond our system are limited considering the relatively small size of our study area and the un-replicated character of our survey. While descriptive studies such as ours, coupled with theoretical predictions, might function as important stepping-stones for research development in this area, they also raise awareness of the issues hindering such progression. To disentangle the community shaping effects of predator control, causal relationships between control intensity and ecological responses must be assessed [26]. Reliable and spatially-explicit measures of intensity and selectivity of the control employed are necessary since different control strategies can lead to contrasting patterns [25,26], thus reducing the utility treatment-control experiments (e.g. [5]) or across studies comparisons (S1 and S2 Tables) when control intensity cannot be described. Similarly, information on spatially-variable control at fine scales, i.e. where culling occurs in the landscape, is needed to assess how carnivore species distribution may be shaped by high mortality areas and potential consequences of such changes in interspecific competition. However, such attempts are severely inhibited by the current practice of illegal and often non-selective killing [32]. Very few studies use direct measures of predator culling intensity, and those that do usually involve qualitative approaches by interviewing game estate managers. Such interviews/surveys often provide only rough estimates and not the needed quantitative information on legal (shooting, trapping) and illegal (snaring, poison) control methods [7,27,82]. Importantly, even for legal predator control, information on trapping effort (e.g. box traps per square kilometre/estate) or the number of animals trapped, for which reporting is mandatory in the framework of permit requirements, is frequently incomplete or absent due to insufficient monitoring and supervision by the responsible entities. This was the reason preventing the use of quantitative data on predator control as covariates in the occupancy analysis in the present study. Information suppression or exaggeration biases, driven by the strong economic and social dimensions surrounding the practice of predator control in small game estates (see review in [7]), represent a major barrier to the development of needed evidence-based approaches to wildlife control, a crucial conflict that resides at the interface of conservation and cultural interests [89].

## Supporting information

S1 Table Species-specific naïve occupancy estimates in the eight studies / nine study areas selected from the literature review. Also includes information on type of management (with or without predator control) and sampling design and the estimates for the present study for comparison. Ss–sampling sites.
(DOCX)

S2 Table Interspecific differences in naïve occupancy between *V*. *vulpes* and sympatric mesocarnivores for each of the eight studies / nine study areas selected from the literature review. Also includes the average difference across species and across studies ± standard deviation. Interspecific differences relative to red fox naïve occupancy obtained in our study were higher than the average value across all species and reviewed studies (average difference = 0.39 ±0.28); with this pattern holding for species-specific average values (across studies) of all mesocarnivores.
(DOCX)

S1 Text Literature review of studies reporting data on Iberian mesocarnivores’ occupancy. (DOCX)
