# Supplementary material for: Mesocarnivore community structure under predator control: Unintended patterns in a conservation context
Source: PLoS One. 2019 Jan 17;14(1):e0210661. doi: 10.1371/journal.pone.0210661 (PMC6336399; doi:10.1371/journal.pone.0210661)
Supplement: S1 Table — Also includes information on type of management (with or without predator control) and sampling design and the estimates for the present study for comparison. Ss–sampling sites. (DOCX) [file pone.0210661.s001.docx]

S1 Table. Species-specific naïve occupancy estimates in the eight studies / nine study areas selected from the literature review. Also includes information on type of management (with or without predator control) and sampling design and the estimates for the present study for comparison. ss – sampling sites.

| **Articles** | **Study area** | **Type of Management** | **Sampling** | ***Vulpes vulpes*** | ***Herpestes ichneumon*** | ***Martes foina*** | ***Mustela putorius*** | ***Mustela nivalis*** | ***Genetta genetta*** | ***Meles meles*** | ***Felis silvestris*** | ***Felis catus*** |
| --- | --- | --- | --- | --- | --- | --- | --- | --- | --- | --- | --- | --- |
| Curveira-Santos et al. (2017) | Portugal | None | 54 ss ~836m apart | 0.95 | 0.80 | 0.13 | 0.04 | - | 0.36 | 0.82 | - | 0.29 |
| Cruz et al. (2015) | Portugal | Unknown | 54 ss ~599m apart | 0.81 | - | 0.90 | - | - | - | 0.67 | - | - |
| Recio et al. (2015) | Spain | Predator control | 37 ss ~2km apart | 0.51 | - | 0.22 | - | - | - | - | 0.24 | 0.11 |
| Barrull et al. (2014) | Spain | Predator control | 75 ss in 1 km^2^ quadrants | 0.50 | - | 0.29 | - | - | - | 0.21 | - | - |
| Monterroso et al. (2014) | Spain | None | 38 ss in 1 km^2^ quadrants | 0.61 | 0.02 | 0.24 | - | - | 0.13 | 0.05 | 0.06 | - |
| Monterroso et al. (2014) | Portugal | Predator control | 32 ss in 1 km^2^ quadrants | 0.22 | 0.15 | 0.26 | - | - | 0.10 | 0.05 | 0.20 | - |
| Sarmento et al. (2011) | Portugal | None | 141 ss. ~472 m apart | 0.53 | 0.13 | 0.34 | - | - | 0.25 | - | - | - |
| Pita et al. (2009) | Portugal | Unknown | 60 ss in 3.14 km^2^ plots | 1.00 | 0.95 | 0.17 | 0.18 | 0.27 | 0.17 | 0.57 | - | 0.75 |
| Barea-Azcón et al. (2007) | Portugal | Unknown | 8 ss in 2.5 km2 plots | 1.00 | - | 1.00 | - | - | 0.38 | 0.25 | 0.38 | - |
|  |  |  | **Average across studies (±SD)** | **0.68 (±0.26)** | **0.41**  **(±0.39)** | **0.39 (±0.30)** | **0.11 (±0.07)** | **0.27** | **0.23 (±0.11)** | **0.37 (±0.29)** | **0.22 (±0.11)** | **0.38 (±0.27)** |
|  |  |  |  |  |  |  |  |  |  |  |  |  |
| **This study** | Portugal | Predator control | 74 ss ~900m apart | 0.92 | 0.10 | 0.35 | - | - | 0.04 | 0.13 | - | 0.38 |
