# Supplementary material for: Mesocarnivore community structure under predator control: Unintended patterns in a conservation context
Source: PLoS One. 2019 Jan 17;14(1):e0210661. doi: 10.1371/journal.pone.0210661 (PMC6336399; doi:10.1371/journal.pone.0210661)
Supplement: S2 Table — Also includes the average difference across species and across studies ± standard deviation. Interspecific differences relative to red fox naïve occupancy obtained in our study were higher than the average value across all species and reviewed studies (average difference = 0.39 ±0.28); with this pattern holding for species-specific average values (across studies) of all mesocarnivores. (DOCX) [file pone.0210661.s002.docx]

S2 Table. Interspecific differences in naïve occupancy between *V. vulpes* and sympatric mesocarnivores for each of the eight studies / nine study areas selected from the literature review. Also includes the average difference across species and across studies ± standard deviation. Interspecific differences relative to red fox naïve occupancy obtained in our study were higher than the average value across all species and reviewed studies (average difference = 0.39 ±0.28); with this pattern holding for species-specific average values (across studies) of all mesocarnivores.

| **Articles** | **Study area** | **Type of Management** | **Sampling** | ***Herpestes ichneumon*** | ***Martes foina*** | ***Mustela putorius*** | ***Mustela nivalis*** | ***Genetta genetta*** | ***Meles meles*** | ***Felis silvestris*** | ***Felis. catus*** | **Average difference across species (±SD)** |
| --- | --- | --- | --- | --- | --- | --- | --- | --- | --- | --- | --- | --- |
| Curveira-Santos et al. (2017) | Portugal | None | 54 ss ~836m apart | 0.14 | 0.82 | 0.91 | - | 0.59 | 0.13 | - | 0.66 | **0.54 (±0.33)** |
| Cruz et al. (2015) | Portugal | Unknown | 54 ss ~599m apart | - | -0.09 | - | - | - | 0.14 | - | - | **0.03 (±0.16)** |
| Recio et al. (2015) | Spain | Predator control | 37 ss ~2km apart | - | 0.30 | - | - | - | - | 0.27 | 0.41 | **0.33 (±0.07)** |
| Barrull et al. (2014) | Spain | Predator control | 75 ss in 1 km^2^ quadrants | - | 0.21 | - | - | - | 0.29 | - | - | **0.25 (±0.06)** |
| Monterroso et al. (2014) | Spain | None | 38 ss in 1 km^2^ quadrants | 0.59 | 0.37 | - | - | 0.48 | 0.56 | 0.55 | - | **0.51 (±0.09)** |
| Monterroso et al. (2014) | Portugal | Predator control | 32 ss in 1 km^2^ quadrants | 0.07 | -0.05 | - | - | 0.12 | 0.17 | 0.02 | - | **0.07 (±0.08)** |
| Sarmento et al.. 2011) | Portugal | None | 141 ss ~472 m apart | 0.40 | 0.19 | - | - | 0.28 | - | - | - | **0.29 (±0.11)** |
| Pita et al. (2009) | Portugal | Unknown | 60 ss in 3.14 km^2^ plots | 0.05 | 0.83 | 0.82 | 0.73 | 0.83 | 0.43 | - | 0.25 | **0.56 (±0.32)** |
| Barea-Azcón et al. (2007) | Portugal | Unknown | 8 ss in 2.5 km2 plots | - | 0.00 | - | - | 0.63 | 0.75 | 0.63 | - | **0.5 (±0.34)** |
|  |  |  | **Average difference across studies (±SD)** | **0.25 (±0.24)** | **0.29 (±0.34)** | **0.86 (±0.07)** | **0.73** | **0.49 (±0.26)** | **0.35 (±0.24)** | **0.37 (±0.28)** | **0.44 (±0.21)** |  |
| **This study** | Portugal | Predator control | 74 camera-traps ~900m apart | 0.82 | 0.57 | - | - | 0.88 | 0.79 | - | 0.54 | 0.72 (±0.15) |
