# Supplementary material for: Mesocarnivore community structure under predator control: Unintended patterns in a conservation context
Source: PLoS One. 2019 Jan 17;14(1):e0210661. doi: 10.1371/journal.pone.0210661 (PMC6336399; doi:10.1371/journal.pone.0210661)
Supplement: S1 Text — (DOCX) [file pone.0210661.s003.docx]

**S1 Text. Literature review of studies reporting data on Iberian mesocarnivores’ occupancy.**

To contextualize our results with previously described patterns we reviewed studies reporting data on Iberian mesocarnivores’ occupancy. We performed a literature search of peer-reviewed scientific articles in the ISI Web of Science (<https://apps.webofknowledge.com>) using as search terms - isolated or combined, within article title, abstract and keywords - the words: “carnivores”, “Iberian Peninsula”, “Portugal”, “Spain”, in conjunction with mesocarnivore species’ common and scientific names. From the resulting list of articles (N=18) we selected only studies with a sampling scale similar to ours, to increase comparability between patterns of carnivore occupancy reported. For each of the selected articles (N=8; 9 study areas), we recorded information on: study area, management typology (with or without predator control), sampling design, and species-specific naïve occupancy values (i.e. the fraction of sampling sites where the species was actually detected).

Selected articles providing data for our analysis were:

1. Barea-Azcón JM, Virgós E, Ballesteros-Duperón E, Moleón M, Chirosa M. Surveying carnivores at large spatial scales: a comparison of four broad-applied methods. Biodivers Conserv. 2007;16: 1213–1230. doi: 10.1007/s10531-006-9114-x

2. Barrull J, Mate I, Ruiz-Olmo J, Casanovas JG, Gosàlbez J, Salicrú M. Factors and mechanisms that explain coexistence in a Mediterranean carnivore assemblage: an integrated study based on camera trapping and diet. Mamm Biol. 2014;79: 123–131. doi: 10.1016/j.mambio.2013.11.004

3. Cruz J, Sarmento P, White PCL. Influence of exotic forest plantations on occupancy and co-occurrence patterns in a mediterranean carnivore guild. J Mammal. 2015;96: 854–865. doi: 10.1093/jmammal/gyv109

4. Curveira-Santos G, Marques TA, Björklund M, Santos-Reis M. Mediterranean mesocarnivores in spatially structured managed landscapes: community organisation in time and space. Agric Ecosyst Environ. 2017;237: 280–289. doi: 10.1016/j.agee.2016.12.037

5. Monterroso P, Rich LN, Serronha A, Ferreras P, Alves PC. Efficiency of hair snares and camera traps to survey mesocarnivore populations. Eur J Wildl Res. 2014;60: 279–289. doi: 10.1007/s10344-013-0780-1

6. Pita R, Mira A, Moreira F, Morgado R, Beja P. Influence of landscape characteristics on carnivore diversity and abundance in Mediterranean farmland. Agric Ecosyst Environ. 2009;132: 57–65. doi: 10.1016/j.agee.2009.02.008

7. Recio MR, Arija CM, Cabezas-Díaz S, Virgós E. Changes in Mediterranean mesocarnivore communities along urban and ex-urban gradients. Curr Zool. 2015;61: 793–801. doi: 10.1093/czoolo/61.5.793

8. Sarmento PB, Cruz J, Eira C, Fonseca C. Modeling the occupancy of sympatric carnivorans in a Mediterranean ecosystem. Eur J Wildl Res. 2011;57: 119–131. doi: 10.1007/s10344-010-0405-x
